# Occurrence of pendelluft under pressure support ventilation in patients who failed a spontaneous breathing trial: an observational study

**DOI:** 10.1186/s13613-020-00654-y

**Published:** 2020-04-07

**Authors:** Andrea Coppadoro, Alice Grassi, Cecilia Giovannoni, Francesca Rabboni, Nilde Eronia, Alfio Bronco, Giuseppe Foti, Roberto Fumagalli, Giacomo Bellani

**Affiliations:** 1 Department of Anesthesia and Intensive Care, San Gerardo Hospital, Monza, Italy; 2 School of Medicine and Surgery, University of Milan-Bicocca, Monza, Italy

**Keywords:** Pendelluft, Electrical impedance tomography, Difficult ventilator weaning, Assisted mechanical ventilation, Spontaneous assisted breathing, Spontaneous breathing trial

## Abstract

**Background:**

Pendelluft, the movement of gas within different lung regions, is present in animal models of assisted mechanical ventilation and associated with lung overstretching. Due to rebreathing of CO_2_ as compared to fresh gas, pendelluft might reduce ventilatory efficiency possibly exacerbating patient’s respiratory workload during weaning. Our aim was to measure pendelluft by electrical impedance tomography (EIT) in patients who failed a spontaneous breathing trial (SBT).

**Methods:**

This is an observational study conducted in a general intensive care unit of a tertiary-level teaching hospital. EIT signal was recorded in 20 patients while pressure support (PS) ventilation was progressively reduced from clinical level (baseline) to 2 cmH_2_O, as in an SBT; four ventral-to-dorsal lung regions of interest were identified for pendelluft measurement. A regional gas movement (> 6 mL) occurring in a direction opposite to the global EIT signal was considered diagnostic for high pendelluft.

**Results:**

Eight patients out of 20 (40%) were classified as high-pendelluft; baseline clinical characteristics did not differ between high- and low-pendelluft patients. At PS reduction, pendelluft and EtCO_2_ increased more in the high-pendelluft group (*p* < .001 and .011, respectively). The volume of gas subject to pendelluft moved almost completely from the ventral towards the dorsal lung regions, while the opposite movement was minimal (16.3 [10:32.8] vs. 0 [0:1.8] mL, *p* = .001). In a subgroup of patients, increased pendelluft volumes positively correlated with markers of respiratory distress such as increased respiratory rate, p0.1, and EtCO_2_.

**Conclusions:**

Occult pendelluft can be measured by EIT, and is frequently present in patients failing an SBT. When present, pendelluft increases with the reduction of ventilator support and is associated with increased EtCO_2_, suggesting a reduction of the ability to eliminate CO_2_.

## Background

Growing evidence suggests that the presence of spontaneous breathing during mechanical ventilation is associated with several advantages, but may be harmful at the same time, particularly during the acute phase [1–3]. One of the possible mechanisms of injury associated with spontaneous breathing and assisted ventilation is the development of pendelluft [1]. Pendelluft is a phenomenon known as the pendular movement of gas between different lung regions; classically it is described during controlled mechanical ventilation when regional heterogeneity in time constants (compliance * resistance) is present: after the tidal volume has been delivered, the gas moves from “faster” lung regions (shorter time constant) towards “slower” ones (longer time constant) [4, 5].

Recently, a different mechanism of pendelluft has been described in animal models and in a small patient series when switching from fully controlled to assisted mechanical ventilation with spontaneous breathing [6]. At the very beginning of inspiration, inflation of the dorsal regions due to uneven distribution of the negative pressure generated by the diaphragmatic pump resulted in a concomitant deflation of the ventral ones, consistent with a movement of gas within the lung (pendelluft). Consequently, this phenomenon may induce overdistension of dorsal regions even in the presence of fully protective “global” ventilator settings. Moreover, the gas subject to pendelluft moving within the lung will not contribute to gas exchange, possibly causing CO_2_ retention, resulting in wasted work of breathing and ventilatory inefficiency.

Switching from controlled mechanical ventilation to an assisted form of ventilation (e.g., pressure support, PS) is a common strategy to progressively increase patient’s work of breathing, for example during weaning [7]. During the weaning phase, patient effort progressively increases until all the inspiratory work is generated by the patient and none by the ventilator. Most patients do not present weaning problems, while others result difficult to wean; prolonged weaning is associated with worst clinical outcomes and increased costs [8–11]. Weaning failure is attributable to different causes, which include respiratory pump insufficiency, cardiovascular dysfunction or underlying infection [12].

**Fig. 1:**
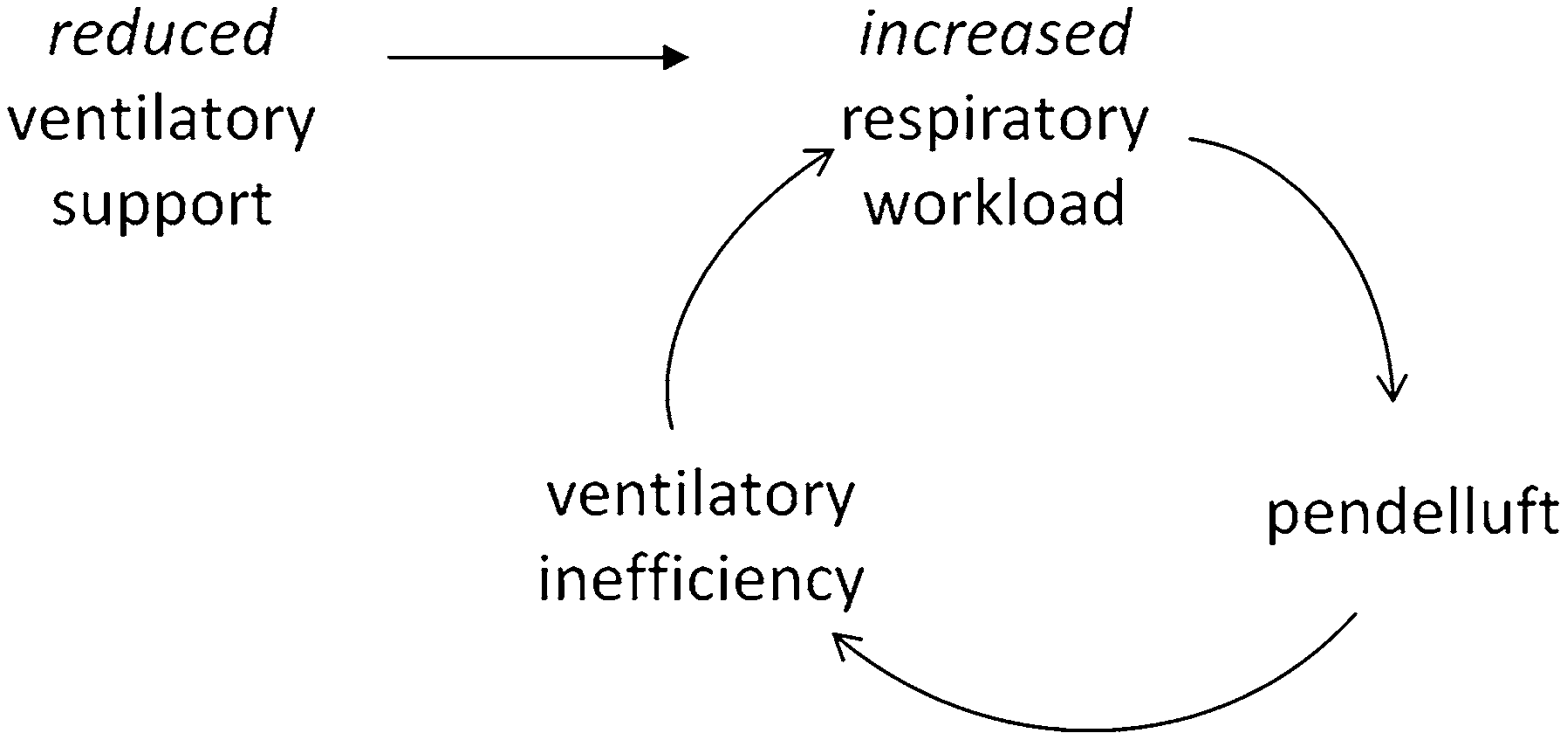
Vicious cycle due to pendelluft during the weaning phase. Illustration of the vicious cycle related with reduction of ventilator support in patients affected by the presence of pendelluft

We speculated that during the decrease of PS, a “vicious circle” might exist in which the increased effort causes pendelluft, thereby decreasing CO_2_ removal and further increasing breathing effort (Fig. 1). The purpose of this work is to assess the presence of pendelluft during the decrease of PS, measuring pendelluft volume and its relationship with clinically relevant variables by electrical impedance tomography (EIT), a non-invasive bedside monitoring technique allowing visualization of regional ventilation which gained increased interest in the recent years [13]. We focused on patients who failed at least one spontaneous breathing trial (SBT) since this population could be more at risk of weaning failure.

## Methods

This is a prospective observational study aimed at assessing the presence of a particular phenomenon such as pendelluft and generating new pathophysiological hypotheses. The local Institutional Review Board (Comitato Etico ASST Monza) approved the study protocol, and written informed consent was obtained as per local regulations.

### Subjects

Patients admitted to the general intensive care unit (ICU) of the University hospital San Gerardo (Monza, Italy) were screened for inclusion criteria: age > 18 years, assisted mode of ventilation (PS is the first choice in our unit), “ready to wean” evaluation by the attending physicians (resolution of acute phase, PEEP ≤ 8 cmH_2_O, FiO_2_ ≤ 40%, light or no sedation, no need for vasopressors), failure of the standard spontaneous breathing trial performed in our ICU (defined as not tolerating PS = 2 cmH_2_O for at least 2 h without signs of respiratory distress). Exclusion criteria were: known severe chronic obstructive pulmonary disease (GOLD 4) [14], pneumothorax, impaired respiratory drive due to cerebral lesions, pneumonectomy, bronchial bleeding, presence of pleural catheters, rib fractures, thoracic wounds, limitations in patient’s mobilization (e.g., spinal fractures); enrollment in other competing study.

### Study protocol

After enrollment, patients were monitored with an EIT system (PulmoVista 500, Dräger Medical GmbH, Lübeck, Germany) and recordings were started at clinically set PS level (baseline); demographic data were collected. PS level was then progressively reduced by 2-cmH_2_O steps, to reach PS = 2 cmH_2_O. All the other ventilator settings were left unchanged during the study as well as ventilator flow trigger. If the patients showed signs of marked respiratory distress, the PS level was increased back to baseline. We collected traces of the EIT signal, and recorded respiratory rate, tidal volume (Tv), Tv distribution by EIT [15], minute ventilation (Mv), mouth occlusion pressure at 100 ms (p0.1) [16], compliance of the respiratory system (C_rs_, measured by inspiratory hold) [17–19], end-tidal carbon dioxide (EtCO_2_), and long-term outcomes such as ICU survival, ICU stay, 28-day ventilator-free days. Rapid shallow breathing index (RSBi) was defined as respiratory rate/Tv [20].

### Pendelluft volume measurement

EIT and ventilator waveforms were analyzed by Labchart, importing data at a 20-Hz rate (ADInstruments, Colorado Springs, CO). Data from a set of 20–30 representative breaths for each PS step were obtained by event-triggered averaging using the first point of positive ventilator airflow as triggering event [21]. Global EIT trace and four regions of interest (ventral-to-dorsal layers of the same thickness, ROI 1–4) were analyzed. The nadir of the global EIT trace was considered as the transition point from expiration to inspiration (*T*_0_); then, the nadirs of the four ROIs were identified together with the values in mL of each ROI at *T*_0_, using the ΔZ of the average tidal volume as a ΔZ/mL converting factor, as previously described [21].

**Fig. 2:**
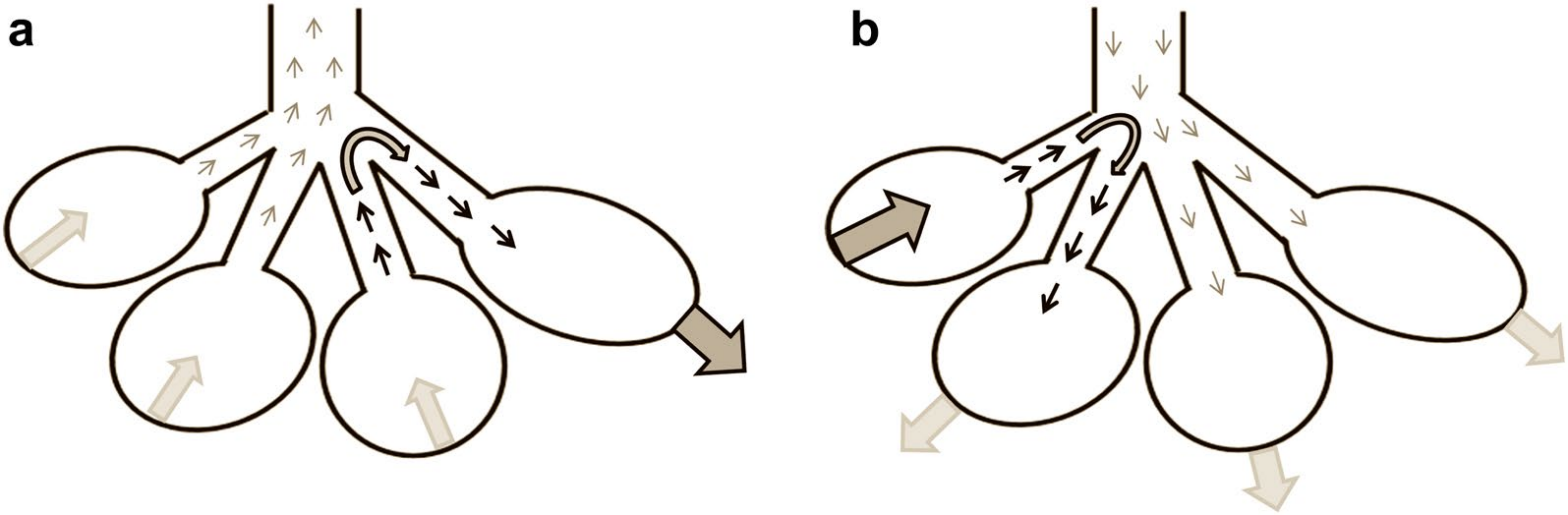
Schematic representation of the pendelluft phenomenon occurring during assisted mechanical ventilation. Pendelluft (black arrows) was defined as the sum of the gas moving into early-inflating regions of interest during expiration (before the global electrical impedance tomography value reached its minimum value, *T*_0_ in the text, panel **a**) and the gas lost by late-deflating regions of interest during inspiration (after *T*_0_, panel **b**)

We reasoned that pendelluft can be disclosed as a phase-shift of the regional EIT signal as compared to the global EIT trace in two distinct time-periods: before *T*_0_ and after *T*_0_. Before *T*_0_, the lung is still expiring and tracheal airflow is directed outward; ROIs inflating during expiration must gain gas from other ROIs that are deflating, indicating the pendelluft phenomenon (Fig. 2, panel a). Conversely, after *T*_0_ tracheal airflow is directed inward, and gas lost by late-deflating ROIs must be gained from the other ROIs that are inflating, indicating the pendelluft phenomenon as well (Fig. 2, panel b). We defined pendelluft volume as the sum of the volumes of gas inspired from the early-inflating ROIs during expiration (before *T*_0_) and the volume of gas expired from the late-deflating ROIs during inspiration (after *T*_0_). Pendelluft volume directed to the ventral lung regions was defined as the sum of gas volume gained by the ventral ROIs before *T*_0_ and the gas volume still to be deflated by the dorsal ROIs after *T*_0_; conversely, pendelluft volume directed to the dorsal lung regions was defined as the sum of gas volume gained by the dorsal ROIs before *T*_0_ and the gas volume still to be deflated by the ventral ROIs after *T*_0_.

Pendelluft volume was compared with regional ventilation delay (RVD), as measured by the software included with the EIT monitor. RVD represents the percentage of lung zones which exhibit a delayed inflation as compared to the global EIT signal (inflation delay > 6% of the global inflating time, see Supplements for further RVD measurement details) [22].

### Statistical analysis

Given the preliminary nature of the study and the lack of data available in the literature regarding incidence of pendelluft in this population, a formal sample size calculation was not performed and we chose a convenience sample size of 20 subjects. Patients were classified as high-pendelluft based on the presence of relevant pendelluft volumes at the lowest PS level; since no defined cut-off exists, we used the mean of the pendelluft volumes measured at baseline (6.3 mL).

Continuous variables were tested for normality by Shapiro–Wilk test. Comparisons between patients were performed by Mann–Whitney or independent sample *t* test; comparisons within the same patient were performed by Wilcoxon or paired-sample t test, as appropriate. Since some patients could not reach the 2-cmH_2_O PS level, changes in respiratory variables were considered at the minimum PS level. Correlations coefficients were expressed as Pearson’s *r*. Comparisons between two categorical variables were performed by Fisher’s exact test. A cut-off of *p* < 0.05 was considered for statistical significance. SPSS was used to perform statistical analyses (SPSS Inc. Chicago, IL).

## Results

The median PS level of the 20 enrolled patients at baseline was 10 cmH_2_O (range 8–12 cmH_2_O); patients’ baseline conditions are reported in Table 1. Patients in the high-pendelluft group were eight, while the remaining 12 were classified as low-pendelluft. At baseline, patients in the high-pendelluft group already showed a pendelluft volume higher than the low-pendelluft group (*p* = 0.003), while the main respiratory variables did not differ. Seventeen patients tolerated the 2-cmH_2_O step for few minutes and recordings were present for all the study steps; one patient in the high-pendelluft and two in the low-pendelluft group developed respiratory distress before the 2-cmH_2_O step, and PS decrement was stopped at 4 cmH_2_O. Comparison between respiratory variables at baseline and at the lowest PS level is reported in Table 2.

**Table 1:** Baseline characteristics of study patients

Baseline data	All patients, *n* = 20	Low-pendelluft group *n* = 12	High-pendelluft group *n* = 8	*p*-value
Age, years	65 ± 9	63 ± 9	68 ± 7	.187
Female sex, n (%)	9 (45)	5 (42)	4 (50)	.535
Body mass index, kg/m^2^	25.5 [22.2–27.4]	25.7 [22–27.7]	25.2 [22.3–27.3]	.791
SAPS2 (ICU admission)	55 ± 19	56 ± 19	52 ± 19	.661
Reason for admission, *n* (%)				.109
Medical	15 (75)	11 (92)	4 (50)	
Emergency surgery	5 (25)	1 (8)	4 (50)	
ARDS, *n*. (%)	12 (60)	9 (75)	3 (37)	.167
Days on CMV	3 [2:7]	3 [1:7]	3 [2:5]	1
ICU days	13 [6:19]	13 [4:27]	13 [12:16]	.970
AMV days	10 [3:14]	12 [2:16]	9 [5:11]	.343
PaO_2_/FiO_2_, torr	253 ± 62	233 ± 60	282 ± 54	.078
PEEP, cmH_2_O	8 [7:8]	8 [7:8]	8 [7:8]	.970
NIF, cmH_2_O	22 ± 11	22 ± 11	21 ± 11	.816
Clinical PSV, cmH_2_O	10 [8:10]	10 [8:10]	9 [8:11]	.624
CPL_rs_, ml/cmH_2_O	48 ± 14	46 ± 12	51 ± 16	.413
Tidal volume, mL	462 [393:541]	487 [335:541]	445 [412:562]	.734
Respiratory rate, bpm	20 ± 7	20 ± 7	20 ± 7	.959
Minute ventilation, L/min	9.5 [6.5:11.5]	8.7 [6.1:10.6]	10.3 [6.5:12.7]	.571
End-tidal CO_2_, mmHg	38 [31:42]	38 [30:54]	38 [31:42]	.762
p0.1, cmH_2_O	1.4 [0.8:2.2]	1.4 [0.9:2.3]	1.2 [0.5:2.1]	.473
RSBi, bpm/L	42 [28:64]	47 [28:65]	38 [27:63]	.537
Tv DEP, %	40 ± 12	37 ± 12	44 ± 12	.188
Pendelluft, mL	3.3 [2.1:8.8]	2.3 [1:3.3]	8.4 [3.7:22.2]	.003
To ventral ROIs, mL	0 [0:0.3]	0 [0:1.3]	0 [0:0.7]	.415
To dorsal ROIs, mL	2.6 [1.4:12.9]	1.6 [0.3:2.8]	7.9 [3.7:22.2]	.002

SAPS2, Simplified Acute Physiology Score II; ICU, intensive care unit; CMV, controlled mechanical ventilation; AMV, assisted mechanical ventilation; PEEP, positive end-expiratory pressure; NIF, negative inspiratory force; PSV, pressure support ventilation; CPL_rs_, compliance of the respiratory system; bpm, breaths per minute; p0.1, occlusion pressure at 100 ms; RSBi, Rapid Shallow Breathing index; Tv DEP, distribution of tidal volume to dependent lung regions. Data are expressed as mean ± SD or median [25th:75th percentile]

**Table 2:** Changes of respiratory variables at the lower pressure support level

Respiratory variable	All patients *n* = 20	Low-pendelluft group *n* = 12	High-pendelluft group *n* = 8	*p*-value
Δ Tidal volume, mL	− 68 [− 123: − 142]	− 56 [-105: − 30]	− 90 [− 158: − 95]	.069
Δ Respiratory rate, bpm	3 [0:7]	2 [0:7]	4 [3:11]	.270
Δ Minute ventilation, L	− 0.2 [− 1.2:0.5]	− 0.3 [− 1.2:0.4]	0 [− 1.6:1.3]	.440
Δ End-tidal CO_2_, mmHg	2 [0:3]	1 [0:1]	3 [2:6]	.011
Δ p0.1, cmH_2_O	1.1 [0.1–2]	1 [0.1–2.1]	1.2 [0.2–1.9]	.910
Δ RSBi, Bpm/L	17 [7-33]	13 [2–30]	23 [12–56]	.190
Δ Pendelluft, mL	1.4 [− 1.3:7.7]	− 0.5 [− 1.7: 1.1]	8.8 [5.6–12.8]	.000
To ventral ROIs, mL	0 [− 1:0]	0 [− 1.3:0]	0 [0:1.1]	.037
To dorsal ROIs, mL	1.5 [0.4:5.8]	0.5 [− 1:1.2]	7.6 [4.7:11.8]	<.001

Bpm, breaths per minute; p0.1, occlusion pressure at 100 ms; RSBi, Rapid Shallow Breathing index; ROI, region of interest. Data are expressed as median [25th:75th percentile]

**Fig. 3:**
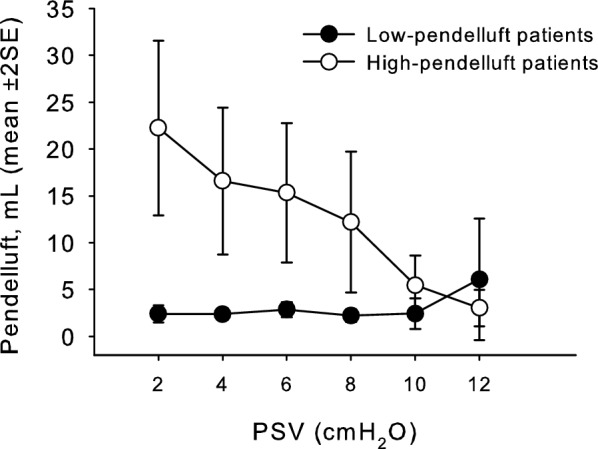
Pendelluft volumes at the reduction of ventilator support. Mean pendelluft volume remained low at the reduction of pressure support ventilation (PSV) level in the low-pendelluft group (filled circles), while increased in the high-pendelluft group (empty circles)

Average pendelluft volumes at the reduction of PS are depicted in Fig. 3. At the lowest PS level, pendelluft volume increased more in the high-pendelluft group, while remained almost stable in the low-pendelluft group (Table 2, Additional file 1: Figure S1 panel a). Similarly, the EtCO_2_ increase was more pronounced in the high- than in the low-pendelluft group at the reduction of ventilator support, and Tv reduction tended to be more marked in the high-pendelluft group (Table 2, Additional file 1: Figure S1 panel b and c). The increase in respiratory rate appeared more pronounced in the high-pendelluft group, but without any statistical significance (Additional file 1: Figure S1 panel d). Pendelluft volumes at the lowest PS level positively correlated with compliance of the respiratory system (*p* = .040 *r* = .463), but not with age, body mass index, SAPS II on admission, days of controlled mechanical ventilation before PS, PaO_2_/FiO_2_ ratio, negative inspiratory force or Tv distribution assessed by EIT; the correlation with compliance resulted significant in the high-pendelluft group (*p* = .014, *r* = .816), but not in the low-pendelluft (*p* = .207, Additional file 1: Figure S2). The average increase of pendelluft volume during reduction of PS levels correlated with average EtCO2 increase (*p* = .044 *r* = .495) and tended to correlate with average respiratory rate increase (*p* = .059 *r* = .429). In the high-pendelluft group, the average increase of pendelluft volume correlated with average increase of heart rate (*p* = .040, *r* = .730).

**Fig. 4:**
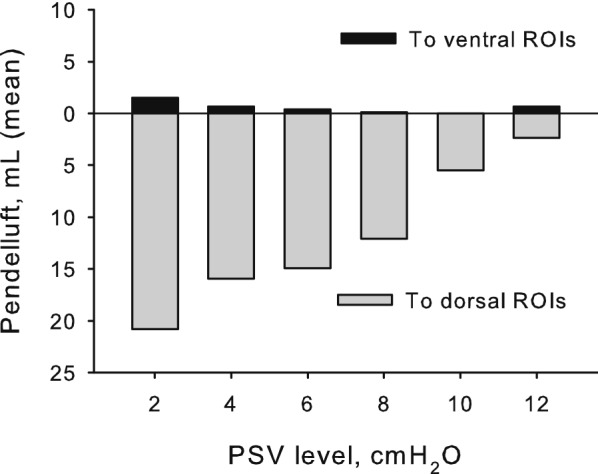
Pendelluft gas movement direction in the high-pendelluft group. In the high-pendelluft group, the movement of gas subject to pendelluft was almost entirely directed towards the dorsal regions of interest, while the amount directed towards the ventral ones was minimal (PSV, pressure support ventilation)

**Fig. 5:**
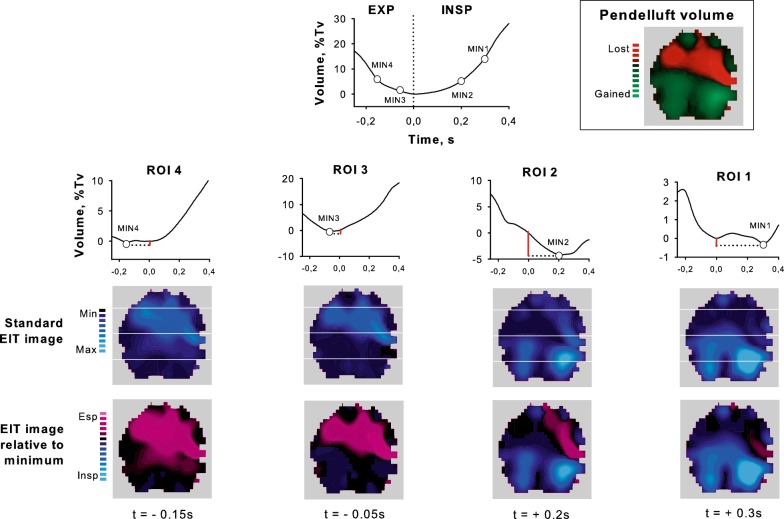
Pendelluft measurement and distribution in patient of the high-pendelluft group (single breath, unfiltered signal) due to early inflation of the dorsal regions and late deflation of the ventral ones. Minimum impedance values of the dorsal regions of interest (ROI 3 and 4) occurred during expiration (EXP), while in the ventral ones (ROI 1 and 2) occurred during inspiration (INSP). Total pendelluft volume was calculated as the sum of the volumes (red bars) inflated during expiration (ROI 3 and 4) and deflated during inspiration (ROI 1 and 2). Processed EIT imaging (relative to minimum) indicated that inspiration in the dorsal ROIs started when ventral ones were still expiring (expiration in purple color); the phenomenon was not immediately evident with standard EIT imaging. Pendelluft gas moved from the ventral towards the dorsal lung regions (boxed image)

**Fig. 6:**
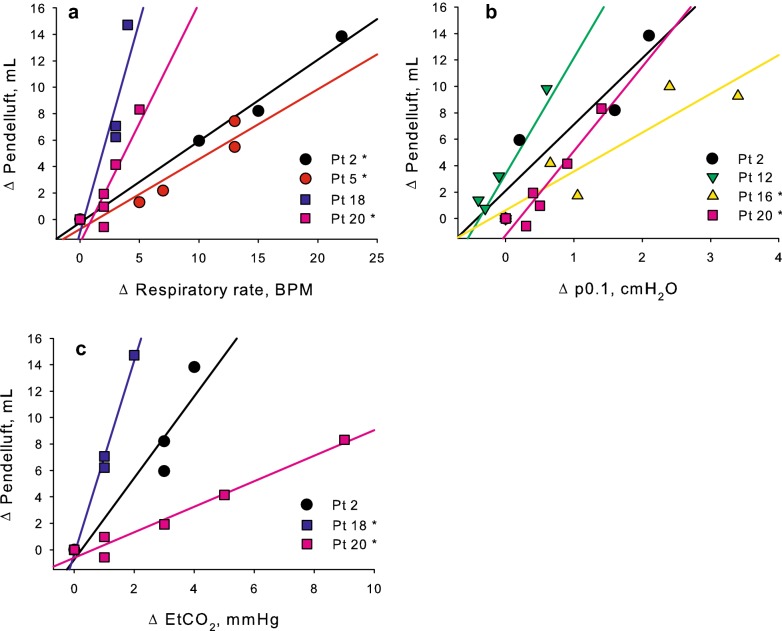
Association between pendelluft volume change during the study and markers of increased inspiratory effort in exemplary patients of the high-pendelluft group. In some patients of the high-pendelluft group, pendelluft volume increase from baseline was associated with markers of increased inspiratory effort such as increased respiratory rate (panel **a**) or increased mouth occlusion pressure at 100 ms (p0.1, panel **b**), or ventilatory inefficiency such as increased end-tidal carbon dioxide (EtCO_2_, panel **c**). Significant correlations are marked with asterisks (*)

Considering the high-pendelluft group, the volume of gas subject to pendelluft moved almost completely from the ventral towards the dorsal lung regions (Figs. 4 and 5, Table 2). Pendelluft volume was unevenly distributed in the four ventral-to-dorsal ROIs, and the pendelluft phenomenon was observed mainly during inspiration rather than at end-expiration (Fig. 2b and Additional file 1: S3). Analyzing within-patient values, in some high-pendelluft patients pendelluft volumes positively correlated with markers of respiratory distress such as the increase of respiratory rate, the increase of p0.1, the increase in EtCO_2_ (Fig. 6).

**Fig. 7:**
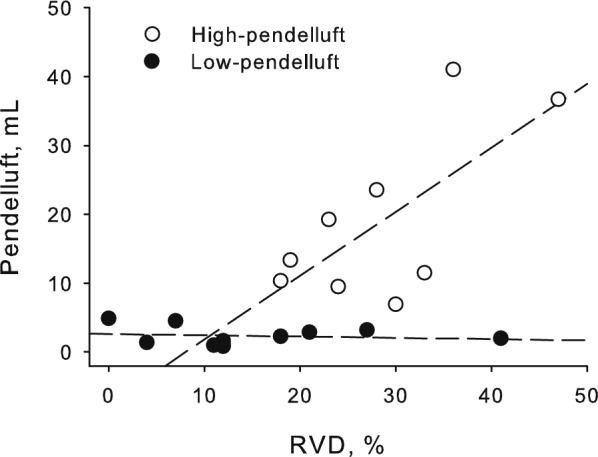
Correlation between pendelluft volume and regional ventilation delay. At the lowest pressure support ventilation (PSV) level, pendelluft volume positively correlated with the percentage of lung zones affected by ventilation delay (RVD) in the high-pendelluft group (*p* = .04, *r* = .69, empty circles), but not in the low-pendelluft group (*p* = .65, filled circles). While in the high-pendelluft group a high regional ventilation delay value was associated with the presence of pendelluft, some patients in the low-pendelluft group showed high regional ventilation delay values but low pendelluft volumes

Pendelluft volumes measured at the lowest PS level positively, albeit loosely, correlated with RVD in the high-pendelluft group (*p* = .04, *r* = .69, RVD range 18–47%), but not in the low-pendelluft group (*p* = .65, RVD range 0–41%, Fig. 7). While in the high-pendelluft group a high RVD value was associated with relevant pendelluft volumes, in the low-pendelluft group high RVD values were present despite the low pendelluft values (representative patient analyses in Additional file 1: Figure S4).

ICU mortality, length of ICU stay and 28-day ventilator-free days did not differ between high- and low-pendelluft groups.

## Discussion

In the present study, we show that pendelluft was present in eight out of 20 patients (40%) and it was possible to detect and quantify it by EIT monitoring. The reduction of PS led to a doubling of baseline pendelluft volume; the phenomenon was almost entirely due to gas movement from the ventral to the dorsal regions. At the lowest PS level, the group of patients with high pendelluft volumes showed a greater increase in EtCO_2_; in a subset of these patients, the increase in pendelluft volume significantly correlated with markers of respiratory distress.

To our knowledge, this is the first clinical study measuring the gas volume subject to pendelluft during spontaneous assisted ventilation, a hidden phenomenon often difficult to recognize from standard ventilator waveforms. The pendelluft phenomenon is classically disclosed in paralyzed patients by an end-inspiratory occlusion maneuver [23]. Recently, EIT availability at the bedside allowed researchers to show the presence of pendelluft during assisted mechanical ventilation: in animal models, a vigorous inspiratory effort caused movement of gas at the beginning of inspiration from ventral to dorsal zones, which are more subject to diaphragm activity, but no quantitative measurement was performed [6]. Of note, none of our patients’ characteristics at baseline was associated with the presence of relevant pendelluft volumes, suggesting that direct monitoring is necessary for its detection. As described in the present study, the pendelluft phenomenon occurred at the moment of cycling from expiration to inspiration. The phenomenon requires a regional monitoring tool with high temporal resolution, making EIT an ideal choice. To remove cardiac-related impedance changes, we used an approach based on event-triggered averaging, a filtering technique that preserves the actual EIT waveform better than standard low-pass filtering [21].

Since gas subject to pendelluft comes from within the lung, it contains higher CO_2_ and lower O_2_ concentrations as compared to fresh gas, and contributes less to pulmonary gas exchange; moving such a gas from one lung region to another results in a waste of energy and reduced ventilatory efficiency, two crucial factors during ventilator weaning. The different response to PS reduction in the high- as compared to the low-pendelluft group and the correlation with markers of respiratory distress in some of the high-pendelluft patients suggest that the chosen cut-off properly identifies the relevance of the phenomenon. However, due to the exploratory nature of our study, we cannot provide any indication about a specific cut-off for future studies.

The detection of pendelluft at baseline and the correlation with respiratory system compliance suggest an association with some intrinsic lung characteristics or with altered respiratory muscles/lung interaction; when workload is increased and respiratory muscles activity is higher, the pendelluft phenomenon directed towards the dorsal zones is then exacerbated. Reduction of PS is associated with redistribution of tidal volume to the dorsal regions, due to higher diaphragmatic activity [15]; we speculate that increased diaphragmatic activity causes pendelluft in some patients at the very beginning of inspiration, leading to an intra-pulmonary movement of gas from the ventral to the dorsal zones, which are more exposed to the diaphragm stretching forces.

While the impact of pendelluft on some respiratory parameters was detectable, the measured pendelluft volumes were relatively small (on average, 5.4% of the Tv at the lowest PS level in the high-pendelluft group, maximum value 8.4%). Previous literature showed that a theoretical pendelluft volume < 2% of the tidal volume can be concentrated in a poorly ventilated region, reaching up to 13% of the volume entering the region and therefore leading to notable effects [4]. Pendelluft volumes were possibly underestimated because the analyzed four ROIs were considered as homogeneous lung zones; the application of smaller ROIs might lead to a higher sensitivity disclosing a higher within-lung movement of gas.

Pendelluft appeared a phenomenon distinct from RVD as known from the literature (i.e., a slower regional inflation as compared to the global signal) [22, 24]. We showed that when pendelluft is present RVD is also present: when a region starts inflation later, it is likely delayed as compared to the entire lung. However, the opposite is not necessarily true, as suggested by the lack of correlation between pendelluft volume and RVD in the low-pendelluft group: some regions may show slow inflation without the presence of pendelluft. Therefore, an RVD measurement tool is not adequate to measure pendelluft, due to excessive false positives.

In the high-pendelluft group, the pendelluft phenomenon was associated with lower ventilatory efficiency and higher distress. While minute ventilation was unchanged, EtCO_2_ increased more in the high-pendelluft group, possibly because of reduced CO_2_ removal due to inefficient intra-pulmonary gas mixture or increased respiratory muscles metabolism [25–27]. Then, a trend towards greater reduction of tidal volumes was recorded, possibly due to a partial wasting of patient’s inspiratory effort for pendelluft gas movement, as described in an obese patient ventilated with insufficient PEEP [28]. Lastly, in some patients the increase in pendelluft volumes positively correlated with markers of respiratory distress such as increased respiratory rate and p0.1. Of note, a higher respiratory rate associated with reduced Tv leads to increased workload, due to higher dead space/tidal volume ratios, further affecting ventilatory efficiency [29]. The reduction of Tv and the increase of respiratory rate are known risk factors for weaning failure; our data raise the hypothesis that pendelluft leading to higher respiratory workload might represent an adjunctive cause of difficulty in weaning [30].

The correlation between the volume of pendelluft and respiratory system compliance might suggest that, in patients with higher compliance, pendelluft could be due to an interaction between diaphragm and lungs with longer (or heterogeneous) time constants, defined as the product of compliance and resistance. In our study protocol, we did not measure airways resistance values. Future studies could elucidate the connection between airway resistance and pendelluft occurrence.

We enrolled patients failing a spontaneous breathing trial reasoning that these patients could be more at risk of difficult weaning and that pendelluft occurrence might be higher in such patients than in easily extubated ones. The restriction of the enrollment to such patients limits the generalizability of the results, but allowed us to focus on subjects for whom even a small increase in ventilatory workload could be relevant. We restricted our investigation to reduction of PS levels; however, during weaning PEEP is also lowered and low PEEP levels are associated with pendelluft exacerbation in animal studies [31]. Future studies might investigate the occurrence of the pendelluft phenomenon in other patient populations, at different PEEP levels and whether pendelluft is to any extent specific to difficult weaning. Another limitation is that our method is based on off-line measurement of pendelluft volumes after EIT data download and analysis; however, the same process could be easily integrated in the current EIT monitors becoming available at the bedside to set the ventilator properly, possibly reducing pendelluft negative effects. Finally, the presence of pendelluft was not associated with relevant long-term outcomes; our study was certainly not powered to disclose such associations, and we believe that long-term outcomes may be explained only in part by single specific factors.

## Conclusions

In our study, EIT technology disclosed the presence of relevant pendelluft volumes during reduction of PS in a fair amount of patients failing a spontaneous breathing trial. Pendelluft was mainly due to the movement of gas from the ventral to the dorsal zone, and appeared associated with increased respiratory workload.

## Supplementary information

**Additional file 1.** A pdf file containing supplemental Methods and Figures (S1–S4).

## Data Availability

The datasets used and/or analyzed during the current study are available from the corresponding author after local Institutional Review Board (Comitato Etico ASST Monza) approval.

## Abbreviations

PS: Pressure support
EIT: Electrical impedance tomography
ICU: Intensive care unit
Tv: Tidal volume
Mv: Minute ventilation
p0.1: Mouth occlusion pressure at 100 ms
C_rs_: Compliance of the respiratory system
EtCO_2_: End-tidal carbon dioxide
ROI: Region of interest
RVD: Regional ventilation delay
