# Supplementary material for: Occurrence of pendelluft under pressure support ventilation in patients who failed a spontaneous breathing trial: an observational study
Source: Ann Intensive Care. 2020 Apr 7;10:39. doi: 10.1186/s13613-020-00654-y (PMC7138895; doi:10.1186/s13613-020-00654-y)
Supplement: Supplementary file 1 — Additional file 1. A pdf file containing supplemental Methods and Figures (S1–S4). [file 13613_2020_654_MOESM1_ESM.pdf]

Additional file 1

Occurrence of Pendelluft under pressure support ventilation in patients who failed a spontaneous breathing trial: an observational study

Andrea Coppadoro, M.D. [1]; Alice Grassi, M.D. [2]; Cecilia Giovannoni, M.D. [2]; Francesca Rabboni, M.D. [2]; Nilde Eronia, M.D. [1]; Alfio Bronco, M.D. [1]; Giuseppe Foti, M.D. [1] [2]; Roberto Fumagalli M.D. [2]; Giacomo Bellani, M.D., Ph.D. [1] [2]

[1] Department of Anesthesia and Intensive Care, San Gerardo Hospital, Monza, Italy

[2] Department of Medicine and Surgery, University of Milan-Bicocca, Monza, Italy

Corresponding author: Giacomo Bellani, Department of Emergency and Intensive Care, San Gerardo Hospital, University of Milan-Bicocca; Via Pergolesi 33, 20900, Monza, Italy; E-mail: giacomo.bellani1@unimib.it; Phone +39 039 233 3293

Materials and Methods

Regional ventilation delay (RVD) measurement

To compare the presence of pendelluft and RVD, RVD was assessed by the software integrated in the PulmoVista electrical impedance tomography (EIT) monitor. The software allows the clinician to obtain the RVD measurement at the bedside by an automatic analysis after selection of 10 consecutive breaths. RVD depends by the fact that different lungs areas may inflate (i.e. reaching the 40% of the total regional inflating volume) with a temporal delay compared to the moment when the global EIT trace reaches the 40% of the total lung inflation. RVD values represent the percentage of ventilated lung inflating more slowly than a specified temporal cut-off. We chose a 6% temporal cut-off: the software marked pixels inflating with a delay >6% of the global inflating time.

Figure S1. Variations of pendelluft volumes and respiratory variables during the study

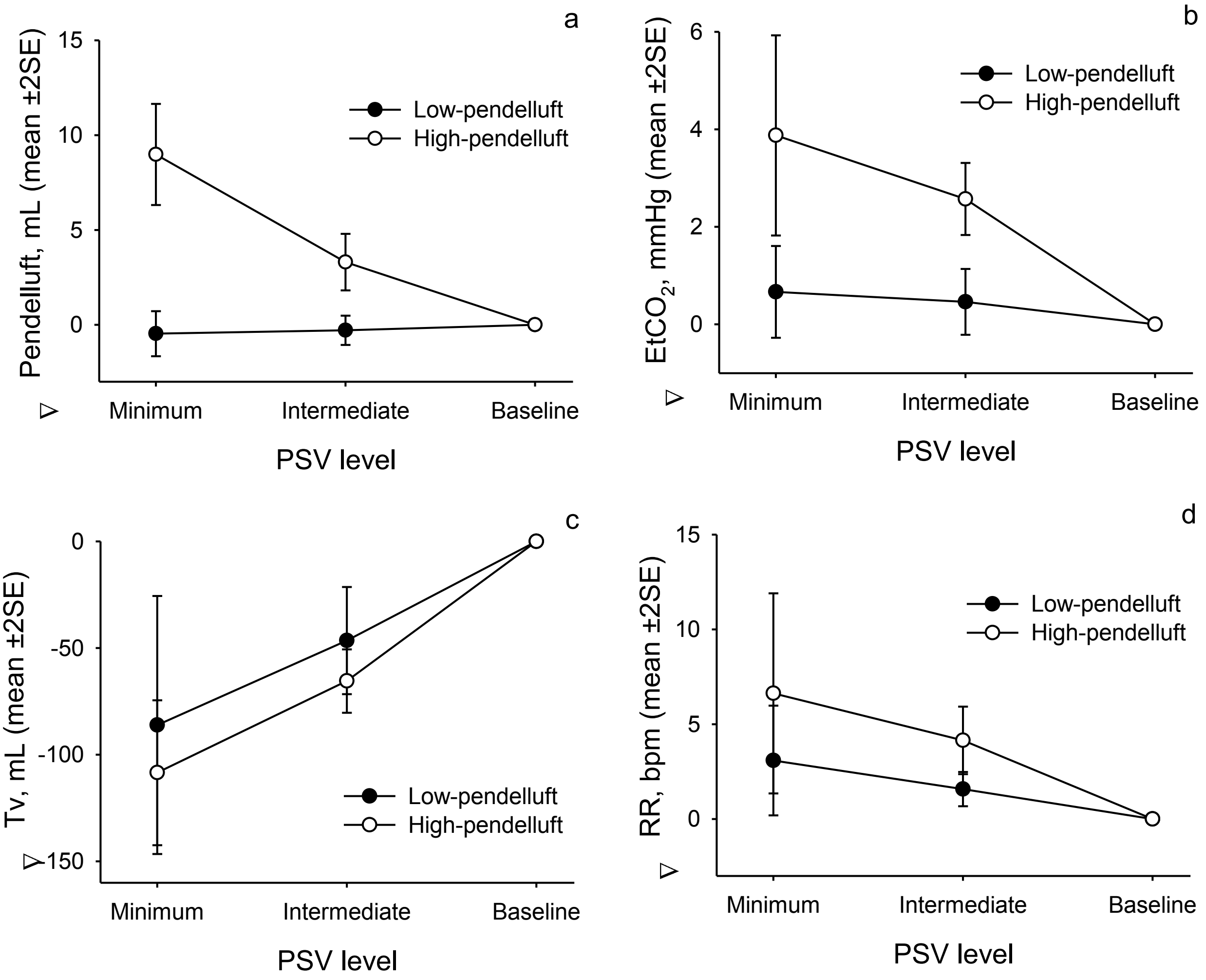

At the minimum pressure support ventilation (PSV) level, the change in pendelluft volume (panel a) and the change in End-tidal Carbon Dioxide ($EtCO_2$, panel b) were higher in the high-pendelluft group as compared to the low-pendelluft group. The change in tidal volumes (Tv, panel c) and respiratory rate (RR, panel d) did not differ (see table 2 for statistics). Intermediate PSV level represents the average values measured during the study, excluding baseline and minimum PSV levels.

Figure S2. Pendelluft volume correlation with respiratory system compliance

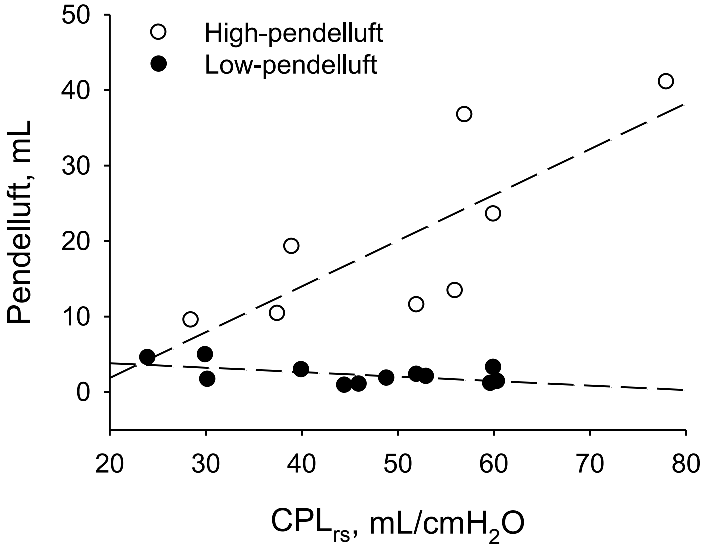

Pendelluft volume at the lowest PSV level positively correlated with compliance of the respiratory system (p=.040 r=.463); the correlation resulted significant in the high-pendelluft group (p=.014, r=.816), but not in the low-pendelluft (p=.207)

Figure S3. Pendelluft volume variations in the 4 studied ventral-to-dorsal ROIs at the reduction of ventilator support in the high-pendelluft group

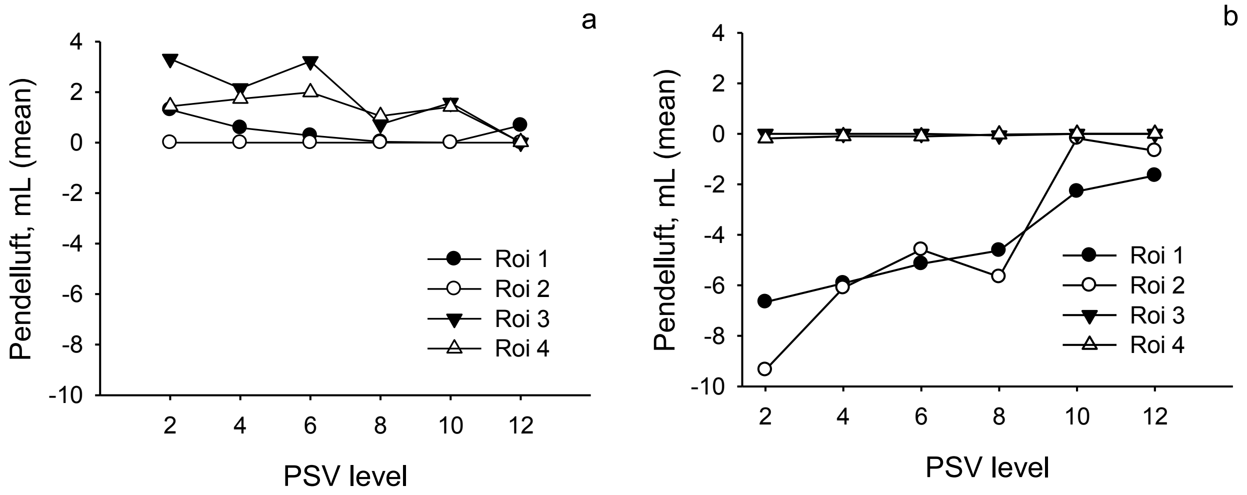

Positive values in panel a represent pendelluft volumes directed *towards* the early-inflating ROIs and occurring before the global electrical impedance tomography (EIT) value reached its minimum value (before inspiration, T_0 in the text), mainly moving to the dorsal lung regions.

Negative values are used in panel b to represent pendelluft volumes *from* the late-deflating regions of interest (after T_0), mainly moving from the ventral lung regions

Figure S4. Regional ventilation delay is not always associated with pendelluft

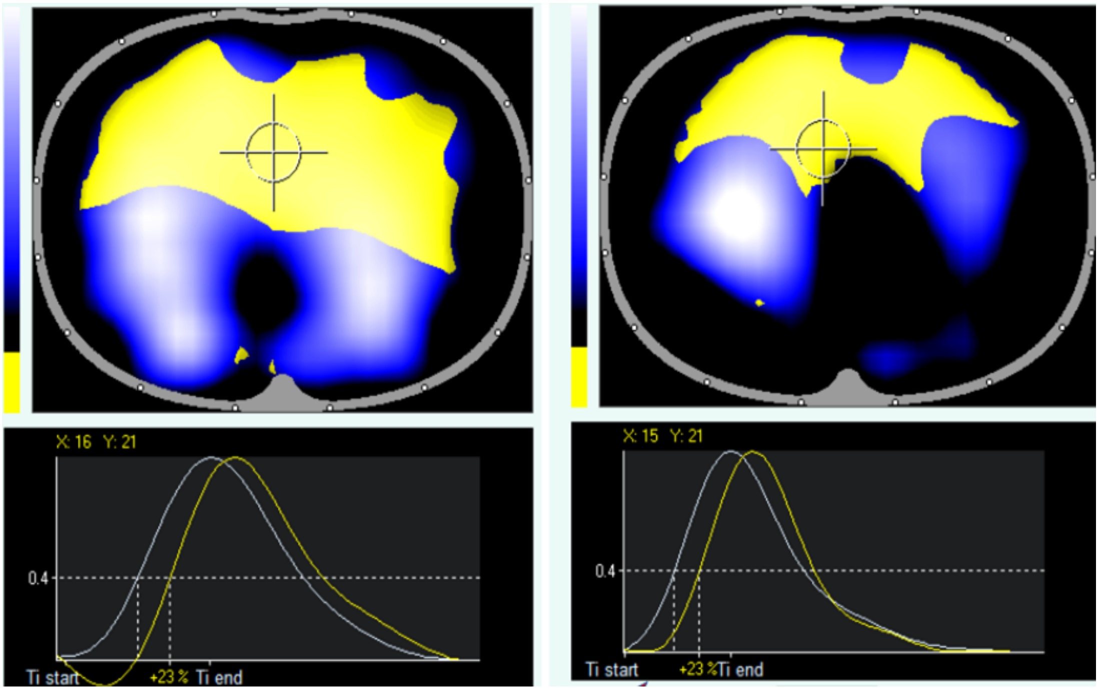

Two representative patients from the high- (left panels) and the low- (right panels) pendelluft groups. A substantial regional ventilation delay (RVD) was measured in both patients (40% and 38% of the ventilated lungs, in yellow in the upper panels). In the high-pendelluft patient, the pixel analysis revealed the presence of pendelluft due to a late deflating lung area (bottom left panel, minimum value of the local yellow trace occurring after the minimum of the global electrical impedance tomography signal, in white). In the low-pendelluft patient, the presence of the same local delay (23%) was not associated with the presence of pendelluft (minimum local and minimum global occur at the same time)
